# Optimization of 2-Acylaminocycloalkylthiophene Derivatives for Activity against *Staphylococcus aureus* RnpA

**DOI:** 10.3390/antibiotics10040369

**Published:** 2021-03-31

**Authors:** Michaelle Chojnacki, Xufeng Cao, Daniel P. Flaherty, Paul M. Dunman

**Affiliations:** 1 Department of Microbiology and Immunology, University of Rochester Medical Center, 601 Elmwood Ave., Rochester, NY 14642, USA; Michaelle_Chojnacki@URMC.rochester.edu; 2 Department of Medicinal Chemistry and Molecular Pharmacology, College of Pharmacy, Purdue University 575 Stadium Mall Dr., West Lafayette, IN 47907, USA; cao309@purdue.edu; 3 Purdue Institute for Drug Discovery, 720 Clinic Dr., West Lafayette, IN 47907, USA; 4 Purdue Institute of Inflammation, Immunology and Infectious Disease, 207 South Martin Jischke Dr., West Lafayette, IN 47907, USA

**Keywords:** *Staphylococcus aureus*, antibiotic, RnpA, mRNA degradation, ptRNA processing

## Abstract

*Staphylococcus aureus* is well-recognized to cause debilitating bacterial infections that are difficult to treat due to the emergence of antibiotic resistance. As such, there is a need to develop new antimicrobials for the therapeutic intervention of *S. aureus* disease. To that end, *S. aureus* RnpA is an essential enzyme that is hypothesized to participate in two required cellular processes, precursor tRNA (ptRNA) maturation and mRNA degradation. Corresponding high throughput screening campaigns have identified the phenylcarbamoyl cyclic thiopenes as a chemical class of RnpA inhibitors that display promising antibacterial effects by reducing RnpA ptRNA and mRNA degradation activities and low human cell toxicity. Herein, we perform a structure activity relationship study of the chemical scaffold. Results revealed that the cycloalkane ring size and trifluoroacetamide moiety are required for antibacterial activity, whereas modifications of the para and/or meta positions of the pharmacophore’s phenyl group allowed tuning of the scaffold’s antimicrobial performance and RnpA inhibitory activity. The top performing compounds with respect to antimicrobial activity also did not exhibit cytotoxicity to human cell lines at concentrations up to 100 µM, greater than 100-fold the minimum inhibitory concentration (MIC). Focused studies of one analog, RNP0012, which exhibited the most potent antimicrobial and inhibition of cellular RnpA activities revealed that the compound reduced bacterial burden in a murine model of *S. aureus* disease. Taken together, the results presented are expected to provide an early framework for optimization of next-generation of RnpA inhibitor analogues that may represent progenitors of a new class of antimicrobials.

## 1. Introduction

*Staphylococcus aureus* is an opportunistic pathogen that resides in the nares of 20–40% of individuals [1,2]. The organism frequently causes disease by exploiting breaches in cutaneous and/or mucosal barriers that may be caused by skin conditions, wounds, or surgical intervention. Resulting, *S. aureus* infections can range in severity from mild skin and soft tissue infections to invasive keratitis, endocarditis, chronic osteomyelitis, pneumonia, and/or bacteremia, resulting in significant morbidity and mortality rates as high as 50% among certain patient populations [3,4,5,6,7]. *S. aureus* disease is highly prevalent. Indeed, the most recent Antimicrobial Surveillance SENTRY Program report found that *S. aureus* was the most common cause of nosocomial bacteremia in North America, and the second most common cause of nosocomial bacteremia in Europe [8]. The organism is also the most common cause of infective endocarditis in the U.S. [9,10] and a predominant cause of ventilator associated pneumonia, which is the most common hospital acquired infection in intensive care units world-wide [11,12,13,14,15]. Moreover, *S. aureus* is the leading Gram-positive pathogen responsible for bacterial keratitis, a condition that leads to approximately 2 million cases of corneal blindness annually worldwide [16,17].

In addition to causing infections frequently, the organism has been exceedingly difficult to treat due to its extraordinary ability to resist the effects of antibiotics, as exemplified by the well-documented emergence- and dissemination- of methicillin resistant *S. aureus* (MRSA) lineages in both healthcare and community settings [18]. More recently, there has been increased prevalence of strains that display intermediate and/or complete resistance to vancomycin, an antibiotic that was once considered a last-line of defense [19,20]. Resistance to more contemporary antibiotics such as daptomycin, ceftaroline, and ceftobiprole have also emerged in recent years [21,22,23,24]. As a result of its widespread ability to resist the effects of antibiotics, *S. aureus* has been designated as one of the six E.S.K.A.P.E. (*Enterococcus faecium*, *Staphylococcus aureus*, *Klebsiella pneumoniae*, *Acinetobacter baumannii*, *Pseudomonas aeruginosa*, and *Enterobacter* spp.) bacterial pathogens of immediate U.S. healthcare concern and for which novel antibiotics are urgently needed [25].

**Figure 1:**
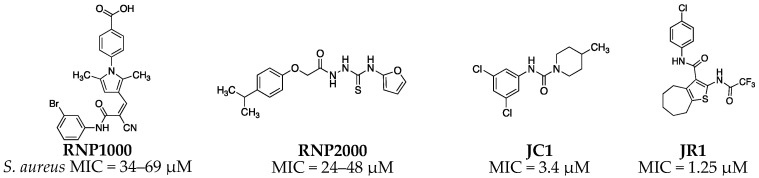
Previously reported RnpA inhibitors with antimicrobial activity against *S. aureus*.

*S. aureus* RnpA is an essential protein that is hypothesized to participate in two required cellular processes, translation and mRNA degradation. Indeed, it is well established that RnpA complexes with the ribozyme *rnpB* to form RNase P enzymes that catalyze the removal of 5′ leader sequences of precursor tRNA (ptRNA) molecules thereby generating mature tRNAs needed for translation [26,27]. More recently, *S. aureus* RnpA has also been found to catalyze the digestion of messenger RNA (mRNA) in vitro in the absence of *rnpB* [28]. Moreover, the protein has been shown to be a component of the organism’s RNA degradosome holoenzyme complex that is thought to modulate cellular mRNA turnover [29,30] and the protein has been structurally characterized [31]. Thus, RnpA may represent a promising novel antimicrobial target that is required for two essential cellular processes and, hence, may provide opportunity to identify small molecule inhibitors of one or both of these essential functions. Accordingly, we previously developed a whole-cell high-throughput mupirocin synergy-based screening approach to screen and simultaneously enrich for library members that display RnpA-associated antibacterial effects [32]. Using this method, several chemical classes of *S.* aureus RnpA inhibitors have been identified and/or characterized, including the aminoglycoside neomycin sulfate [32], RNPA1000 [28], RNPA2000 [33], and the recently identified JC and JR series of compounds [34] (Figure 1).

With regard to the JR chemical class of phenylcarbamoyl cycloalkylthiopene RnpA inhibitors, members of this chemical class have been established to display potent RnpA-dependent anti-staphylococcal activities and limited or no detectable human cell cytotoxicity [34]. Early structure-activity relationship (SAR) studies designed to probe the pharmacophore’s phenyl moiety established that variations in the ring are tolerated for anti-staphylococcal activity and primarily affected RnpA’s ptRNA processing function. Herein, we describe results of expanded SAR studies that are designed to further evaluate the scaffold for RnpA inhibitory and anti-staphylococcal activities.

## 2. Results

### 2.1. Chemistry

**Scheme 1:**
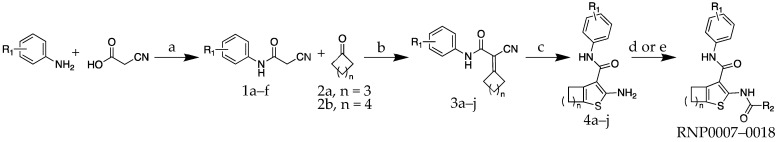
Synthesis of RnpA inhibitor analogs RNP0007-0018. Reagents and conditions: (a) R_1_-substituted aniline (1 eq), 2-cyanoacetic acid (1 eq), EDC-HCl (1.2 eq), DCM, rt, overnight; (b) 1a–f (1 eq), 2a or b (7 eq), NH_4_OAc (5 eq), AcOH (7 eq), Na_2_SO_4_ (5 eq), toluene, 100 °C, 2 h; (c) 3a–j (1 eq), S_8_ (2 eq), morpholine (3 eq), EtOH, reflux, 4 h; (d) for analogs RNP0007–0016: 4a-j (1 eq), trifluoroaceticanhydride (1.2 eq), triethylamine (1.2 eq), DCM, rt, overnight; (e) for analogs RNP0017–0018: 4a (1 eq), R_2_-CO_2_H (1.2 eq), EDC-HCl (1.25 eq), 4-DMAP (0.1 eq), rt, overnight.

The molecules were all synthesized using a procedure similar to one reported by Truong et al. [35] As shown in Scheme 1, the first step was to use substituted aniline reagents to form a set of substituted-cyanoacetamide intermediates 1a–f. These were then coupled with a cyclic ketone as part of a Knoevenagel condensation reaction to form intermediates 3a–j. The Knoevenagel products were carried directly into a cyclization step using octasulfur and morpholine to produce 2-aminothiophene intermediates 4a–j. The 2-aminothiophenes were finally coupled with either trifluoroacetic anhydride or a fluoroacetic acid reagent to yield the final compounds RNP0007–RNP0018 to be used in the antimicrobial and in vitro assays.

### 2.2. Antimicrobial Structure-Activity Relationship of 2-acylcycloalkylthiophene Analogs

Analogs were first assessed for antimicrobial activity against *S. aureus* strain UAMS-1 to understand the SAR for the scaffold as it pertains to antimicrobial activity (Table 1). As previously reported, minimum inhibitory concentration (MIC) testing revealed molecule JR1 exhibited single-digit micromolar antimicrobial activity (MIC = 1.25 µM). [34] First, SAR was probed on the phenyl ring at R_1_. Moving the chlorine atom from the *para-* to the *meta*-position improved antimicrobial activity by approximately threefold to 0.391 µM for RNP0007. Swapping out the chlorine for a fluorine in RNP0008 provided no change in activity as the MIC value remained at 0.391 µM. The preference for the 3-substituted halogen was reiterated by the fluorine analogs as the 4-F derivative was slightly less potent.

Switching from a single halogen on the phenyl ring to a methyl (RNP0010) actually reduced activity by eightfold to an MIC value of 3.215 µM compared to the 3-F counterpart, RNP0008. Interestingly, the activity was restored when the methyl was converted to the trifluoromethyl derivative in RNP0011 with an MIC value of 0.391 µM. To round out the SAR study on the phenyl group, the CF_3_-substituent was moved to the *para*-position in RNP0012 and proved to be more potent than the *meta*-substituted nearest neighbor analog with an MIC of 0.19 µM.

The next four analogs investigated the role of the cycloalkane ring size on anti-staphylococcal activity. In these molecules the cycloalkane ring was contracted from a seven-membered ring to a six-membered ring. Across the board these six-membered ring analogs displayed a significant reduction of antimicrobial activity compared to the seven-membered ring matched molecular pairs. For example, the 4-CF_3_ containing six-membered cycloalkyl thiophene derivative RNP0015 was fourfold less potent than the corresponding seven-membered ring counterpart RNP0012. The same reduced activity based on ring size was more prominent for the fluorophenyl analogs as both the six-membered cycloalkyl ring containing molecules only exhibited MIC values of 6.25 µM, while the seven-membered ring derivatives had MIC values as low as 0.391 µM (RNP0008). Thus, it was clear among this analog set that ring contraction was detrimental to antimicrobial activity regardless of the substituent at R_1_. However, the SAR trend observed for the phenyl substitution with the seven-membered cycloalkyl ring did carry over to the six-membered ring analogs. This is illustrated by the fact that the 4-CF_3_ version at R_1_ was the most potent among each cohort lending further confidence the observed SAR was indeed tractable.

Finally, modifications to the trifluoroacetamide moiety on the 2-amine of the thiophene were explored. The trifluoroacetamide is actually a common protective group for amine functional groups and can be labile under both low and high pH conditions. However, the stability at the low pH condition, i.e., pH ≤ 1, requires high heat to cleave the amide bond. Thus, even though it has been reported that the trifluoracetamide to be stable under normal physiological conditions [35] we sought to assess analogs that systematically removed fluorine atoms. To this end we found that any modification to the trifluoroacetamide functional group, both the di- and mono-fluoroderivatives (RNP0017 and RNP0018 respectively), were completely inactive against *S. aureus* at concentrations up to 500 µM. This result is consistent with analogs from our previous manuscript on the scaffold in which the trifluoroacetamide was replaced with either an acetamide or the 2-aminothiophene was left unsubstituted and both analogs also displayed significantly reduced activity against *S. aureus* [34]. Combined these analogs all highlight that the trifluoroacetamide appears to be critical to the antimicrobial activity observed for the scaffold.

To summarize, for single halogen substitution at R_1_ the molecules preferred 3-substition for enhanced antimicrobial activity over the 4-position. However, this trend was flipped when the larger trifluoromethyl moiety was appended to R_1_ as the 4-position was preferred. Cycloalkyl ring size off the thiophene also heavily affected antimicrobial activity with the seven-membered ring being the most effective. Finally, the trifluoroacetamide was shown to be critical for antimicrobial activity consistent with our previous findings. The most potent analog of the set was RNP0012 with an MIC = 0.19 µM, which contained a seven-membered cycloalkyl ring and R_1_ = 4-CF_3_. This molecule represents a 6.6-fold improvement over the previous best in this class JR1. It also is more effective than the previously reported RNPA2000, [33,36] which represents a different chemical series of RnpA inhibitors.

### 2.3. In Vitro RNA Metabolism Activity for Analogs

To assess whether improvements and/or reductions in each analog’s antibacterial performance were associated with RnpA inhibitor activity, in vitro RnpA mRNA degradation assays were performed in the presence and absence of each analog, as previously described [33,34].

#### 2.3.1. In Vitro mRNA Degradation Activity

**Figure 2:**
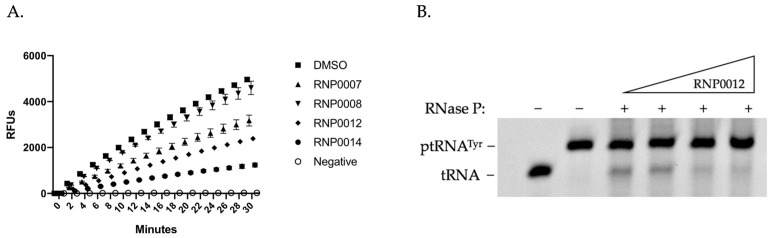
Representative RNP analog inhibition of in vitro RnpA-mediated RNA degradation (**A**) or ptRNA^Tyr^ processing (**B**). Panel A. DMSO (◼☐) or 50 µM of compound was incubated with RnpA in the presence of the FRET RNA substrate for 30 min at 37 °C. Fluorescence measurements (RFUs) were collected every 2 min at excitation 490 nm, emission 520 nm. The negative control (**○**) with no protein ensures the absence of contaminating nucleases. Panel B. 0, 50, 250, or 500 µM RNP0012 was incubated with 10 pmol RNase P and ptRNA^Tyr^ for 30 min at 37 °C and conversion of ptRNA to tRNA was evaluated by urea-PAGE; migration of tRNA^Tyr^ and ptRNA^Tyr^ are shown.

As shown in Figure 2A for representative analogs, a time course study revealed that while RnpA efficiently degraded the RNA substrate in reactions performed in presence of DMSO (squares) reactions performed in the presence of 50 µM of either of the compounds RNP0007, 8, 12, or 14 displayed varying degrees of RNA degradation inhibition. Subsequent dose-response assays for each analog to determine the IC_50_ value provided further resolution of the inhibitory effects of each compound (Table 2). Results revealed that compounds RNP0007, 8, 9, and 10, which displayed excellent anti-staphylococcal activity, were the least effective at inhibiting RnpA-mediated RNA degradation, raising the possibility that their antibacterial effects could be modulated by the inhibition of RnpA-depended ptRNA processing or by affecting another cellular target(s). In contrast, RNP0014 which exhibited reduced antibacterial activity in comparison to the parent (JR1), displayed the lowest IC_50_ and was the strongest inhibitor of RNA degradation. Compounds RNP0017 and 18 exhibited excellent in vitro RnpA RNA degradation activity (IC_50_ values of 43 µM and 33 µM, respectively), despite having no detectable antibacterial activity, suggesting perhaps an inability of the compounds to gain entry to the cell. The remaining compounds had generally good inhibitory activity, with IC_50_ values ranging from 43 to 68 µM, yet the overarching trend from these studies was that while alterations of the three regions of the scaffold affect the series antimicrobial activity the changes do not correlate strongly with an inhibition of RnpA in vitro RNA degradation function.

#### 2.3.2. In Vitro RnpA ptRNA Processing Activity of Analogs

Next, we evaluated each analog’s ability to limit the RnpA’s in vitro precursor tRNA processing activity, as previously described [33]. To do so, RNase P (RnpA + *rnpB*) was reconstituted and combined with ptRNA^Tyr^ and the proteins ability to facilitate the conversion of precursor tRNA^Tyr^ to mature tRNA^Tyr^ was measured in the absence and presence of increasing concentrations of each compound. Figure 2B shows representative data of the performance of RNP0012 in which increasing concentrations of the analog reduced conversion of precursor tRNA to mature tRNA in a dose dependent manner. All analogs were tested at least twice and their average IC_50_ values were recorded (Table 2). Compound RNP0015, which displayed improved antibacterial performance in comparison to JR1, exhibited the greatest inhibitory activity (IC_50_ value), whereas compounds RNP007, 14, and 17, all displayed weaker ptRNA processing inhibition in the range of >95 µM. Compound RNP0017, which did not appear to display any inhibition of ptRNA processing also did not exhibit any antibacterial activity. Likewise, RNP0014, which also displayed reduced antibacterial activity in comparison to JR1, exhibited weak effects on RnpA-mediated ptRNA processing despite having excellent inhibition of mRNA degradation activity. The remaining compounds RNP0008, 9, 10, 11, 12, 16 and 18 had moderate inhibitory effects in vitro, ranging in IC_50_ values between 52 and 79 µM.

Taken together results of in vitro mRNA degradation and ptRNA processing revealed that analogs RNP0011, 12, 13, 15, 16, and 18 all had good inhibitory properties of both functions and RNP0007 weakly inhibited both activities. RNP0008, 9, and 10 only inhibited the ptRNA processing function, whereas 13, 14, and 17 had weak or no inhibitory activity towards RnpA-mediated ptRNA processing. RNP0017 and 18 exhibited inhibitory activity of RnpA mRNA activity in vitro but had no detectable antibacterial activity in the whole cell MIC testing, suggesting that the trifluoroacetamide moiety may be important for cell entry or inhibition of a second unknown target. Ultimately, for this class there was limited correlation between either in vitro assay or the antimicrobial activity.

### 2.4. Activity of RnpA Inhibitors on RNA Metabolism in Cellular Environment

Given that there was limited correlation between the in vitro RNA processing experiments and the antimicrobial properties of the molecules we reasoned that there may be disparities between how the in vitro assays represent the in cellulo properties of enzymes as they relate to the chemical class. To investigate this possibility we performed bacterial cell-based assays of RnpA mediated mRNA degradation and ptRNA processing for each analog, as previously described [34].

#### 2.4.1. Mupirocin Synergy Activity of Each Analog

As a first step toward evaluating the cellular RnpA inhibitory activity of each analog, we returned to our mupirocin synergy screening assay to assess whether each compound displays synergy with mupirocin. The premise for this assay is that antimicrobial compounds, such as sulfonamides and trimethoprim, that inhibit independent steps in an essential pathway often display improved antibacterial activity in combination [37,38]. In that regard, the antibiotic mupirocin inhibits isoleucyl tRNA synthetase upstream of RnpA-dependent RNase P in the translation pathway and previous studies have revealed that RnpA inhibitors are additive/synergistic in combination with mupirocin [32,34]. Fractional inhibitory concentration (FIC) testing revealed that, as expected, most analogs exhibited either additive (RNP0007-13 and 16) or synergistic (RNP0014) antimicrobial effects, with FIC index values falling between 0.5 and 1 (Table 3), suggesting the antimicrobial effects observed may be RnpA dependent. Importantly, none of the analogs exhibited synergistic activity in combination with antibiotics targeting other cellular pathways (data not shown).

#### 2.4.2. Inhibition of Intracellular mRNA Turnover

**Figure 3:**
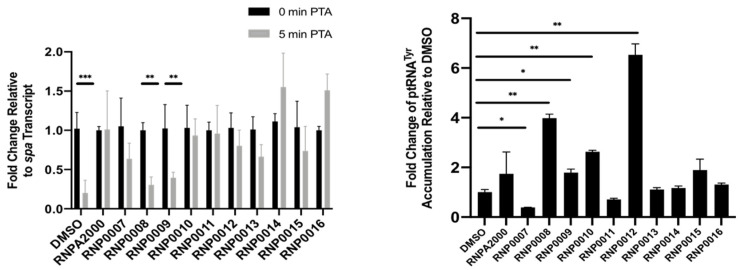
Quantitative RT-PCR measures of *spa* transcript turnover (left) and ptRNA^Tyr^ accumulation (right) in *S.aureus* UAMS-1 cells treated with DMSO, JR analogs or RNPA2000 (positive control). For *spa* RNA turnover measures, cells were treated with 0.5X MIC of each compound for 30 min prior to transcriptional arrest. For ptRNA^Tyr^ accumulation studies, cells were treated 0.5X for 1 h. Each compound was evaluated twice, and values averaged. * *p*-value < 0.05, ** *p*-value < 0.01, *** *p*-value < 0.0001 (Two-way ANOVA).

As a more direct measure of whether the antimicrobial effects were RnpA-dependent, studies were performed to measure their inhibition of cellular RnpA-mediated mRNA turnover of *spa* mRNA. The *spa* mRNA is a natural substrate of RnpA mRNA degradation, as previously described [28,33,34], and can be utilized for an intracellular readout of inhibition for RnpA-mediated mRNA turnover. To do so, *S. aureus* cells were treated with a sub-inhibitory concentration of compound (0.5X MIC) then challenged with the antibiotic rifampicin to arrest de novo RNA synthesis. Quantitative RT-PCR was used to measure the cellular titers of *spa* mRNA at 0 and 5 min post-transcriptional arrest. As shown in Figure 3 (left) cells treated with DMSO (negative control) rapidly degraded *spa* mRNA within 5 min of transcriptional arrest, whereas cells treated with RNPA2000 (positive control) displayed reduced cellular mRNA degradation. The majority of the analogs inhibited cellular mRNA turnover compared to DMSO control, with RNP0014 and RNP0016 being the strongest stabilizers of mRNA. RNP0008 and 9 were the weakest inhibitors of cellular RNA turnover, which was also observed in vitro (Table 2). Surprisingly, RNP0010 was a strong inhibitor of cellular mRNA turnover despite having weak inhibitory activity in vitro. RNP0011 and 12 were moderate inhibitors of RnpA-mediated RNA turnover.

#### 2.4.3. Inhibition of Intracellular prRNA Processing

To evaluate the effects of each analog on cellular RnpA-mediated ptRNA processing, *S. aureus* cells were treated with a sub-inhibitory concentration of each analog (0.5X MIC) and qRT-PCR was used to measure ptRNA^Tyr^ accumulation, as previously described [33,34]. As shown in Figure 3 (right), DMSO-treated cells did not accumulate ptRNA^Tyr^, while treatment with the positive control, RNPA2000, resulted in an increase in ptRNA^Tyr^ accumulation, as expected. Compounds RNP0007, 11,13, 14, 15, and 16 had little effect on ptRNA^Tyr^ levels in the cellular context. The largest increases in ptRNA^Tyr^ levels were measured in cells treated with compounds RNP0008, 9, 10, and 12, all of which were considered highest priority analogs as they displayed both in vitro inhibitory activity and antimicrobial activity.

Taken together, all of the analogs evaluated displayed inhibitory activity against one or both RnpA-mediated RNA metabolism functions. It was observed that RNP0012 exhibited the greatest combinatory effect against each process, and this translated to this molecule displaying the greatest antimicrobial potency against *S. aureus*.

### 2.5. Evaluation of Analogs for Cytotoxicity on Human Cells

**Figure 4:**
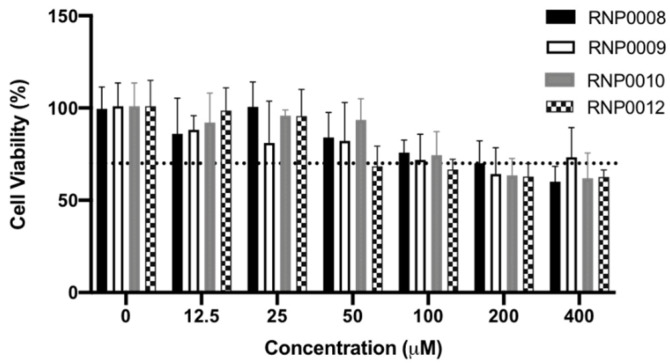
Effects of prioritized compounds on human cell viability. RNP0008 (black), RNP0009 (white), RNP0010 (grey), and RNP0012 (checkered) were added to HEPG2 cells in increasing concentrations (*x*-axis). The dashed line represents the lower limit of tolerable cell viability, below which compounds are considered to have toxic effects. Values are an average of two experiments performed in triplicate.

For the scaffold to have translational significance, it must be non-toxic to human cells. Accordingly, an initial assessment of toxicity was performed using human liver epithelial (HEP G2) cells via a 3-(4,5-dimethylthiazol-2-yl)-2,5-diphenyltetrazolium bromide (MTT) assay in the presence of up to 400 µM of each analog. Figure 4 provides representative viability data from the series of analogs that displayed the greatest antimicrobial and cellular RnpA inhibition, whereas data for all analogs is provided in Table S1. Mitomycin C was used as a positive control at 400 µM, which resulted in a decrease to 54.8% viability (Table S1). Viability of 70% was used as the cut-off criteria for toxicity (Figure 4, dashed line), according to ISO guidelines, as described in Materials and Methods.

Of the prioritized molecules, RNP008, 9, and 10 did not exhibit toxicity that was beyond the 70% cut-off criteria at concentrations lower than 50 µM. RNP0012 displayed 68% viability against HEP G2 cells at this dose, which is 263-fold above the MIC against *S. aureus* for this analog. Survey of all of the analogs (Table S1) revealed that most compounds were only moderately toxic at 50 µM with viability ranging from 70 to 93% compared to untreated cells. RNP0013 displayed the greatest toxicity at 52.9% cell viability at the highest doses of 200 and 400 µM. Taken together, these data indicate that the molecules are non-toxic at the reported MICs, and generally have low toxicity at concentrations up to 400 µM.

### 2.6. Evaluation of RNP0012 Efficacy in a Murine Model of Bacterial Keratitis

**Figure 5:**
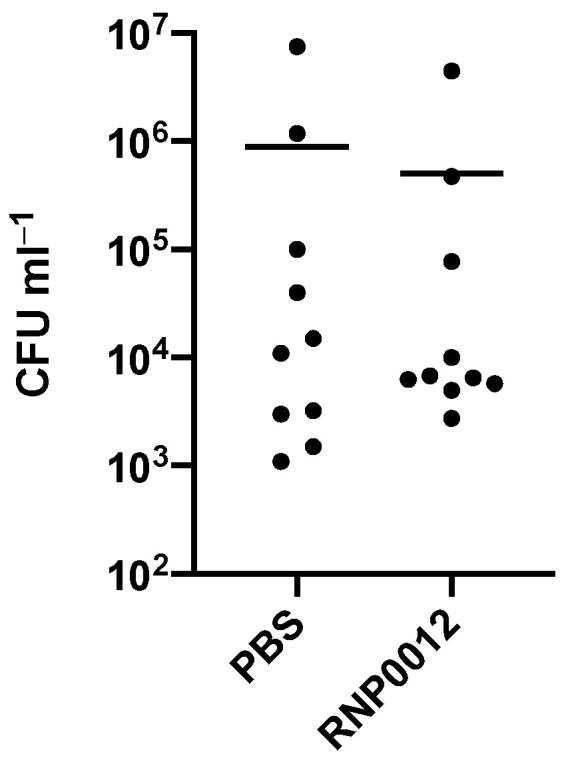
Efficacy of RNP0012 in a murine model of bacterial keratitis. Corneal scratches were inoculated with 10^7^ CFU mL^−1^ UAMS-1 and dosed with PBS (n = 10) or 50 µM RNP0012 (n = 9) every 6 h for 48 h.

Finally, in a pilot study to assess the therapeutic potential of the chemical series RNP0012 was selected for in vivo efficacy in a murine keratitis model of *S. aureus* infection [39] because the analog displayed the combination of the most potent anti-staphylococcal activity, low toxicity, and ability to inhibit both RnpA-mediated cellular functions within bacterial cells. Corneal infections were induced by inoculating corneal scratches with *S. aureus*, eyes were treated with RNP0012 6 h later and every 6 h thereafter for 48 h and bacterial burden was enumerated. Mice treated with RNP0012 displayed a 1.2-log CFU reduction in comparison to vehicle treated animals (Figure 5). While the reduction observed in this pilot study was not considered statistically different (*t*-test), the promising trend indicates that RNP0012 formulation and/or dosing optimization may lead to a new strategy for treating *S. aureus* keratitis.

## 3. Discussion

*S. aureus* is well-recognized for its ability to cause severe bacterial infections that lead to high rates of morbidity and mortality. While historically considered an opportunistic nosocomial pathogen, the recent emergence of hypervirulent strains that are capable of causing community-associated infections combined with the organism’s ability to resist front-line antibiotics and nonsensical outright elimination of most pharmaceutical antibacterial development groups have further exacerbated healthcare concern.

To that end, one of our major goals has been to explore the therapeutic promise of strategies that are designed to exploit *S. aureus* RnpA for antimicrobial development purposes. The premise for doing so is that the protein is hypothesized to be a unique antibacterial target that is required for two essential cellular processes, ptRNA processing and mRNA degradation [33]. As such, it is possible, even likely, that resistance to corresponding RnpA inhibitors may be slow to develop as target site mutations that limit inhibitor binding may be tolerated by one of the protein’s activities but limit the other. Moreover, the development of such antimicrobials may be accelerated from the perspective that they may achieve therapeutic antimicrobial efficacy as a function of synergy associated with partially crippling two cellular activities as opposed to requiring the laborious task of developing exquisite inhibitors of a “single” target molecule to achieve efficacy.

Accordingly, small molecule inhibitors of *S. aureus* RnpA-associated mRNA degradation (RNPA1000), ptRNA processing (neomycin) and both functions (RNPA2000) have been identified [28,32,33]. In keeping with the dual essentiality hypothesis and desire to develop compounds that effect both RnpA mRNA and ptRNA processing activities, medicinal chemistry campaigns designed to explore RNPA2000′s therapeutic promise and reduce the scaffold’s chemical liabilities have revealed that while one can successfully modify the chemical series furan moiety, eliminating its central thiourea group results in a loss of RnpA inhibition and anti-staphylococcal activity, reducing enthusiasm for the scaffold’s development [36]. More recently, two additional chemical scaffolds, JC and JR1, have been identified has potent RnpA inhibitors with excellent antibacterial activity [34]. The goal of the current study was to perform an early JR1 structure activity relationship. To do so, 12 analogs were synthesized to probe the scaffold’s phenyl ring, cycloalkane ring size, and trifluoroacetamide moieties, with each compound evaluated for antimicrobial activity, mammalian cytotoxicity, in vitro and cellular RnpA-dependent ptRNA and mRNA effects.

From these studies, SAR was further developed for the 2-acylaminocycloalkylthiophene scaffold that will help guide optimization of future next-generation analogs. More directly, results revealed that the seven membered cycloalkane ring size is important, as restriction to a six-membered ring significantly reduced antimicrobial activity. Indeed, comparisons of matched molecular pairs revealed that reducing the ring size limited antimicrobial activity between 4- and 16-fold. In vitro assays revealed that the reduced ring size compounds appeared to retain, or improve, RnpA inhibitory activity of some compounds, suggesting that the enhanced antibacterial activity of the seven-membered ring is attributable to bacterial compound entry and/or intracellular stability and exemplifies the importance of cell-based assays in establishing a SAR. Similarly, results also revealed that the trifluoracetamide moiety is required for antimicrobial activity, as any modifications to this group appeared to completely eliminate bacterial activity, which is consistent with our previous finding that its removal radically reduced antibacterial performance [34]. Exploration of the scaffold’s phenyl ring revealed RnpA dependent antimicrobial tunability. Here, changes of the *para*-Cl to F improved antimicrobial activity, which was further enhanced by switching to a trifluoromethyl group, resulting in compound RNP0012 with a 6.6-fold increase in anti-staphylococcal activity (MIC 0.19 µM). Modifications of the *meta* position also were well tolerated, suggesting that future exploration of the scaffold’s *meta-* and *para-* positions, both alone and in conjunction, are warranted.

In addition to excellent antimicrobial activity, RNP0012 exhibited little mammalian cytotoxicity (therapeutic index of ~260) and displayed among the highest inhibition of in vitro RnpA mediated mRNA degradation and ptRNA processing activities. Likewise, it was a top performer in cellular assays of RnpA-dependent mRNA degradation and ptRNA processing. More specifically, RNP0012 was additive in combination with mupirocin, but not other antibiotics, providing an initial indication that the compound’s antimicrobial activity is RnpA-dependent. Indeed, the compound significantly reduced RnpA-associated mRNA degradation within *S. aureus* cells and was the top-performing cellular ptRNA processing inhibitor among all analogs evaluated. For these reasons we focused our attention on using RNP0012 in a pilot study of the scaffold’s therapeutic usefulness.

*S. aureus* is a predominant cause of bacterial keratitis, which is an invasive infection of the cornea and contributes to approximately 2 million worldwide cases of blindness annually [17]. A corresponding *S. aureus* keratitis model has been developed in our laboratories that allows an early indication of antibiotic efficacy in the absence of complicating factors such as PK/PD associated with more traditional systemic models of delivery [39]. In a pilot study using this model we found that scratched murine corneas inoculated with *S. aureus* show a 1.2 log reduction in bacterial burden in comparison to vehicle treated animals. While the treatment did not reach statistical significance, the trend certainly indicates that the reduction in bacterial burden can be further enhanced by higher concentration, more frequent dosing, or a refined/optimized RNP0012 next-generation analog or formulation. Consequently, the 2-acylaminocycloalkylthiophene chemical series may represent a promising new class of RnpA targeting antimicrobials worthy of further exploration.

## 4. Materials and Methods

### 4.1. Chemistry

#### General Experimental

^1^H, ^13^C, and ^19^F NMR spectra were recorded on Bruker DRX500 or ARX-800 spectrometers in [D_6_] DMSO or CDCl_3_ with or without the internal standard of TMS at 0.05 or 0.1% *v/v*. The purity of all final compounds was >95% purity as assessed by HPLC. Final compounds were analyzed on an Agilent 1200 series chromatograph. The chromatographic methods used were either (A) ThermoScientific Hypersil GOLD C18 column (3 µM particle size, 150 mm length, 4.6 mm ID) or (B) ThermoScientific Hypersil GOLD C18 column (3 µM particle size, 250 mm length, 4.6 mm ID). UV detection wavelength = 254 nm; flow rate = 1.0 mL min^−1^; solvents: (A) water (0.1% trifluoroacetic acid *v/v*) and (B) acetonitrile (0.1% trifluoroacetic acid *v/v*). Gradient: 5–95% solvent B over 9 min [40]. The mass spectrometer used is an Advion CMS-L Compact Mass Spectrometer with an ESI or an APCI ionization source. Samples are submitted for analysis using either the atmospheric solids analysis probe (ASAP) or flow injection analysis (FIA). Compounds were prepared according to the following protocols and are detailed in the Supplementary Material.

### 4.2. Biological Evaluation

#### 4.2.1. Bacterial Growth Conditions

*Staphylococcus aureus* strains UAMS-1 is a well-characterized antibiotic susceptible osteomyelitis clinical isolate (Blevins Infect. Immun 2003). For experiments, UAMS-1 was cultured in Mueller Hinton (MH) broth and processed as described below. *Escherichia coli* strain BL21 (DE3) was cultured in Luria–Bertani (LB) and supplemented with ampicillin (50 µg ml^−1^) where indicated.

#### 4.2.2. Antimicrobial Susceptibility Testing

Minimum inhibitory concentration (MIC) was performed according to the Clinical and Laboratory Standards Institute (CLSI) guidelines. Briefly, individual wells of a 96-well microtiter plate were inoculated with 10^4^ colony forming units (CFU) of *S. aureus* UAMS-1 containing two-fold increasing concentrations (0 µM to 100 µM) of the indicated antibiotic or putative RnpA inhibitor and grown at 37 °C for 16 h in MH broth. The MIC was defined as the lowest concentration of compound in which there was no visible bacterial growth detected by the unaided eye.

Fractional inhibitory concentration (FIC) testing was performed, as previously described [41]. Briefly, each well of a 96-well microtiter plate was inoculated with 10^4^ CFU of *S. aureus* UAMS-1 [42]. Each row of the plate was supplemented with two-fold increasing concentrations of mupirocin (0 µg mL^−1^ to 2 µg mL^−1^) whereas each column contained two-fold increasing concentrations of putative RnpA inhibitor compound (0.03, 0.0625, 0.125, 0.25, 0.5, 1, or 2× MIC). Following bacterial inoculation, plates were incubated at 37°C for 16 h in MH broth and the MIC was determined by the unaided eye. The FIC index was defined by the following formula: FIC = (MIC of Drug A in Combination/MIC of Drug A Alone) + (MIC of Drug B in Combination/MIC of Drug B Alone). A synergistic interaction was defined as a FIC value of ≤0.5, an additive interaction as 0.5–1, no interaction as 1–4 and antagonistic interaction as FIC ≥ 4, as described [41].

#### 4.2.3. RnpA Protein Purification

His-tagged RnpA was purified as previously described [33]. *E. coli* BL21 (DE3) cells harboring plasmid pEXP5-nt containing a hexahistidine tag fused to the N-terminus of *S.aureus* RnpA coding region were grown in LB supplemented with 50 µg mL^−1^ ampicillin to OD_600_ = 0.6 and induced with 1mM isopropyl β-D-1-thiogalactopyranoside (IPTG) for three hours to induce protein expression. *E. coli* cells were subjected to centrifugation at 1560× *g* for 20 min at 4 °C and the cell pellet was resuspended in 20 mL buffer A (300 mM NaCl, 50 mM Na_2_HPO_4_, pH 7.4) containing a mini EDTA-free protease inhibitor tablet (Roche; Bradford, CT, USA) and 20 mM imidazole. Cells were mechanically lysed by three passes at 18,000 psi through a French Pressure Cell Press (SLM-Aminco; Pittsford, NY, USA) and cell debris was removed by centrifugation at 17,000× *g* for 10 min at 4 °C. Supernatants were collected, filtered through a 0.2 micron syringe filter, then loaded onto a 5 mL HisPur cobalt column (ThermoFisher Scientific, Waltham, MA, U.S.A.) using the BioRad Maximizer Duo-Flow Medium Pressure Chromatography System. Protein was eluted using an imidazole gradient (80 mM to 500 mM). Fractions were assessed for RnpA presence and purity via SDS-PAGE analysis, Coomassie staining and Western blotting using an anti-His antibody (Invitrogen; Grand Island, NY, USA).

#### 4.2.4. In Vitro Transcription of RNA

*rnpB* and RNA substrates (*spa*, ptRNA^Tyr^) for RnpA functional assays, as well as mature tRNA were synthesized in vitro, as previously described [33]. Each gene was PCR amplified using *S. aureus* UAMS-1 chromosomal DNA as a template and the corresponding oligonucleotide primer pairs, in which the forward primers contained an RNA polymerase T7 promoter sequence. The following primer pairs were used in this study (T7 promoter underlined): T7-*rnpB* forward—GATTACATAATACGACTCACTATAGGGTGATATTTCGGGTAATCGCTATA and *rnpB* reverse—ACTAGTAGTGATATTTCTATAAGCCATG; T7-ptRNA^Tyr^ forward—GATTACATAATACGACTCACTATAGGGCACCATTTATGGAGGGGTAGCG and ptRNA^Tyr^ reverse—TGGTGGAG GGGGGCAGATTC; T7-*spa* forward—GATTACATAATACGACTCACTATAGGGTTATAGTTCGCGA CGACGTCCAG and *spa* reverse—TTGAAAAAGAAAAACATTTATTCAATTCGTAAACTAGG. Resulting PCR products were analyzed on a 0.8% agarose gel and purified using the Qiagen QIAquick Gel Extraction kit, according to the manufacturer’s instructions. In vitro transcription was performed using the TranscriptAid T7 High Yield Transcription kit (Fermentas; Burlington, ON, Canada) following manufacturer’s instructions. Following in vitro transcription, the RNA was treated with DNase I for 90 min and re-purified using the Qiagen RNeasy Mini kit. RNA was quantified using a NanoDrop2000 spectrophotometer.

#### 4.2.5. In Vitro mRNA Degradation Assays

RnpA-mediated degradation was assessed using the Rnase Alert QC System (Thermofisher Scientific). 20 pmol of His-RnpA was combined with 0.4 μM (final concentration) FRET substrate and either DMSO (negative control) or compound ≤500 µM in 1× Rnase Alert Buffer. Reactions were assembled in 96-well PCR plates (BioRad) and incubated at 37 °C for 30 min in the BioRad CFX 96 Connect Real Time instrument, measuring fluorescence every 2 min at 490 nm excitation/520 nm emission. Percent inhibition of each compound was calculated at the 30 min time-point based on the following formula: (RFU of compound treated RnpA/RFU of DMSO-treated RnpA) × 100.

#### 4.2.6. In Vitro ptRNA Processing Assays

*S.aureus* Rnase P activity assays were performed as previously described [33]. Briefly, ptRNA^Tyr^ and *rnpB* RNA was denatured by heating to 95 °C for 5 min then slow cooled to room temperature. RNA species were then combined with 2× low salt buffer (50 mM Tris-HCl pH 8.0, 5 mM MgCl_2_) and incubated for 5 min at 37 °C. Rnase P was reconstituted by mixing an equal molar ratio of His-RnpA and *rnpB* for 15 min at 37 °C. Precursor tRNA processing inhibition reactions were performed by combining 5 pmol of reconstituted Rnase P with DMSO (negative control) or 50 µM compound and incubating for 5 min at 37 °C. Then, 5 pmol ptRNA^Tyr^ was added to each reaction and incubated for an additional 30 min at 37 °C. Reactions were stopped with 2× RNA loading dye (Thermo Scientific) and heating to 65 °C for 10 min. Samples were electrophoresed on a 7M urea/8% polyacrylamide gel then stained with 0.5 µg mL^−1^ ethidium bromide. The BioRad EZ Gel Doc imaging system was used to visualize the RNA and relative abundance of mature tRNA^Tyr^ in the DMSO control or in the samples containing compound and analyzed using BioRad Image Lab densitometry software. The percent inhibitory activity of each compound was calculated using the following equation: (% experimental processing/% processing negative control) × 100.

#### 4.2.7. Cellular mRNA Turnover Assays

*S.aureus* RNA half-life measurements were performed as described [33]. Briefly, *S.aureus* UAMS-1 was grown to an optical density at 600 nm of 0.18 in MHB and treated with DMSO (negative control) or 0.5× MIC of the indicated compound for 30 min with shaking at 37 °C. To inhibit de novo RNA synthesis, rifampin (Alfa Aesar, Haverhill, MA, USA) was added to the culture at a final concentration of 200 µg mL^−1^. Half of the culture was immediately removed and combined with an equal volume of ice-cold acetone:ethanol (1:1 *v/v*). The remainder of the culture was returned to the 37 °C incubator for an additional 5 min, then combined with an equal volume of ice-cold acetone:ethanol (1:1 *v/v*) and stored at −80 °C.

#### 4.2.8. Cellular tRNA^Tyr^ Population Measures

Cellular *S. aureus* tRNA^Tyr^ pools were measured as previously described [34]. Briefly, *S. aureus* was grown to OD_600_ = 0.18 in MHB and treated with DMSO (negative control), 0.5× or 1× MIC of the indicated compound for 1 h with shaking at 37 °C. The culture was removed, combined with an equal volume of acetone:ethanol (1:1 *v*/*v*) and stored at −80 °C.

#### 4.2.9. Bacterial RNA isolation and Quantitative Reverse Transcription Polymerase Chain Reaction (qRT-PCR)

Cell suspensions in acetone:ethanol were thawed on ice and centrifuged at 1560× g for 10 min at 4 °C. Acetone:ethanol was decanted, and pellets were left to air-dry for 5 min. Cell pellets were resuspended in 500 µL ice-cold TE buffer (10 mM Tris-HCl, pH 8.0, 1mM EDTA) and transferred to FastPrep Lysing Matrix B tubes (MP Biomedicals, Santa Ana, CA). Mixtures were homogenized at 5 m∙s^−1^ for 20 s, rested on ice for 5 min, then homogenized again at 4.5 m∙s^−1^ for 20 s in a FastPrep-24 instrument. Cell lysates were centrifuged at 16,200× *g* for 15 min at 4 °C to remove cell debris. Total bacterial RNA and tRNA^Tyr^ populations were isolated from the supernatant using Qiagen RNeasy Mini kits and miRNeasy kits, respectively, following the manufacturer’s recommendations (Germantown, MD). For qRT-PCR, 2 µg of RNA substrate was treated with 2 units of DNase I (New England Biolabs, Ipswich, MA, USA) for 1 h at 37 °C and re-purified using Qiagen RNeasy Mini kits, following the manufacturer’s instructions. RNA was measured for concentration and quality using a NanoDrop spectrophotometer. Quantabio (Beverly, MA, USA) qScript cDNA Supermix was used to convert 200 ng of RNA into cDNA, following manufacturer’s instructions, which was subsequently amplified using Quantabio SYBR Green Fast Mix following manufacturer’s instructions. Fluorescence was read on the BioRad (Hercules, CA, USA) CFX 96 Connect Real Time Machine. Transcript levels were compared to the internal control 16S rRNA (ΔΔCt) and plotted as fold change compared to the control. The following primer pairs were used in this study for qRT-PCR: *spa* forward—GCAGATAACAAATTAGCTGATAAAAACAT; *spa* reverse—CTAACGCTAATGATAATCCACCAAATAC; –15 ptRNA^Tyr^ forward—TTAACTGAATAAGCTGGAGGGG; tRNA^Tyr^ reverse—TGGTG GAGGGGGGCAGATTC; 16S rRNA forward—TAACCTACCTATAAGACTGGGATAA; 16S rRNA reverse—GCTTTCACATCAGACTTAAAAA.

#### 4.2.10. Cytotoxicity Testing

Human liver epithelial cells (HEP G2) were cultured in Dulbecco’s Modified Eagle Medium (DMEM; Fisher Scientific, Hampton, NH) supplemented with 10% heat inactivated fetal bovine serum (FBS; Corning Life Sciences) and 1% penicillin/streptomycin (Thermofisher Scientific). Cells were incubated at 37 °C with 5% CO_2_ in Nunc (Roskilde, Denmark) tissue culture flasks. Cells were grown in monolayers until exceeding 70% confluency, removed with 0.25% trypsin (Fisher Scientific), resuspended in fresh medium, and used to seed approximately 2.5 × 10^5^ cells/mL into each well of a 96 well tissue culture microplate (Nunc) containing 200 µL of fresh medium. Cytotoxicity testing was conducted according to the guidelines of the International Organization for Standardization (ISO) 10993-5:2009. After 24 h of incubation, the cell media was removed, and adherent cells were washed with 1× phosphate buffered saline (PBS). Cell media was supplemented with 5% per volume of compound at final concentrations from 12.5 µM to 400 µM and incubated 20–24 h at 37 °C with 5% CO_2_. After incubation, medium supplemented with compound was removed from the wells and replaced with 100 µL of fresh culture medium. Then, 10 µL of 12 mM (3-(4,5-dimethylthiazol-2-yl)-2,5-diphenyltetrazolium bromide (MTT) stock reagent (CyQUANT MTT cell proliferation kit; Thermofisher Scientific) was added to each well. After labeling the cells with the MTT reagent, all but 25 µL of medium was removed from the wells, and 50 µL of DMSO was added to each well and mixed thoroughly and cells were incubated at 37 °C with 5% CO_2_ for 10 min. After incubation, wells were mixed again and using a SPECTRAmax5 microplate reader, absorbance was read at 540 nm. Cells were treated with 125 µg/mL mitomycin C (Fisher Scientific) to serve as a positive control and 2% DMSO as a negative control. Following ISO guidelines, compounds that resulted in cell viability below 70% were considered to have cytotoxic effects. All compounds were tested in in two independent experiments in triplicate and cell viability was expressed as a percent of the negative control.

#### 4.2.11. Murine Corneal Infection Model and Treatment

In vivo efficacy studies utilizing a murine model of corneal *S. aureus* infection were performed as previously described [39]. Female BALB/c mice four to six weeks of age were acquired from Charles River (Washington, MA, USA) and housed according to the approved University of Rochester Medical Center Council on Animal Research (UCAR) protocol. *S. aureus* clinical isolate USA300 was grown overnight on both MH agar and MH broth at 37 °C. Broth cultures were centrifuged at 2000× *g* for 10 min at 4 °C and the pellet was resuspended in 1× sterile PBS in a volume equivalent to the starting volume. The mice were anesthetized with 100 mg kg^−1^ ketamine (Par Pharmaceutica; Chestnut Ridge, NY, USA) and 10 mg kg^−1^ xylazine (Akorn, Inc.; Lake Forest, IL, USA) and 0.5% proparacaine (Akorn, Inc.) was applied to the right eye of each mouse. Excess proparacaine was blotted from the ocular surface. Using a 27-gauge needle, inoculated with a single colony of *S. aureus* USA300, three 2 mm scratches were made across the right cornea. The scratches were inoculated with a 5 µL volume of the bacterial culture containing 10^7^ CFU. After infection, the eyes were assigned a disease severity score every 24 h, using a previously validated scale, as follows: 0, no evidence of clinical infection; 1, opacity < 4 mm; 2, opacity > 4 mm; 3, dense opacity covering the entire cornea; 4, perforation of the cornea (Beisel and Berk 1983). In the event of a perforation, the mouse was euthanized immediately via carbon dioxide asphyxiation. Treatments were administered in 5 µL aliquots to the infected eyes every 6 h beginning 6 h post-inoculation and ending 48 h post-inoculation. The treatments included PBS (negative control) and RNP0012, diluted in PBS to 50 µM. The animals were euthanized after 48 h and whole eyes were excised, homogenized, and plated to quantitate viable *S. aureus* colonies present. To do so, eyes were added to tubes containing 1.4 mm ceramic beads (Fisher Scientific; Hampton, NJ, USA) and 0.5 mL PBS and homogenized using the Fisherbrand Bead Mill homogenizer. Aliquots of 0.1 mL were removed from each tube, serially diluted in 0.8% sodium chloride, and plated on mannitol salt agar plates (Fisher Scientific) for enumeration after incubating 16 h at 37 °C, any animals that appeared.

## 5. Conclusions

This study describes work designed to study the structure activity relationship of the 2-acylaminocycloalkylthiophene chemical class of RnpA inhibiting antimicrobials. In doing so we found features of the chemical class, such as the cycloalkane ring size and trifluoroacetamide moiety, were indispensable for antimicrobial activity toward *S. aureus*. Conversely changes to the scaffold’s phenyl ring were well tolerated and modulated RnpA targeting effects, which were best measured in cell-based assays of the protein’s mRNA degradation and ptRNA activities. A frontrunner, RNP0012 was identified to exhibit excellent antimicrobial activity and inhibition of both RnpA functions that appeared to display appreciable antimicrobial performance in a murine model of *S. aureus* keratitis.

## Figures and Tables

**Table 1:**
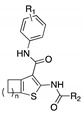
Antimicrobial activity of analogs against *S. aureus.*

Compound	R_1_	R_2_	n	MIC (µM)	MIC (µg/mL)
RNPA2000	-	-	-	44	15
JR1 ^a^	4-Cl	CF_3_	4	1.25	0.52
RNP0007	3-Cl	CF_3_	4	0.391	0.16
RNP0008	3-F	CF_3_	4	0.391	0.16
RNP0009	4-F	CF_3_	4	0.78	0.32
RNP0010	3-CH_3_	CF_3_	4	3.125	1.24
RNP0011	3-CF_3_	CF_3_	4	0.391	0.18
RNP0012	4-CF_3_	CF_3_	4	0.19	0.086
RNP0013	3-F	CF_3_	3	6.25	2.4
RNP0014	4-F	CF_3_	3	6.25	2.4
RNP0015	4-CF_3_	CF_3_	3	0.78	0.34
RNP0016	3-CF_3_	CF_3_	3	3.125	1.36
RNP0017	3-Cl	CHF_2_	4	>500	>200
RNP0018	3-Cl	CH_2_F	4	>500	>200

All minimum inhibitory concentration (MIC) values determined using *S. aureus* strain UAMS-1, ^a^ Values reported by Colquhoun et al. [34].

**Table 2:** MIC and in vitro inhibition values for RNA metabolism.

Compound	MIC (µM)	mRNA IC_50_ (µM)	ptRNA IC_50_ (µM)
**RNPA2000**	44	130	130
**JR1 ^a^**	1.25	50	7
**RNP0007**	0.391	175	98
**RNP0008**	0.391	245	78
**RNP0009**	0.78	>250	79
**RNP0010**	3.125	224	70
**RNP0011**	0.391	59	57
**RNP0012**	0.19	68	66
**RNP0013**	6.25	50	95
**RNP0014**	6.25	6.4	138
**RNP0015**	0.78	43	46
**RNP0016**	3.125	45	52
**RNP0017**	>500	43	>250
**RNP0018**	>500	33	58

^a^ Values reported by Colquhoun et al. [34].

**Table 3:** Fractional inhibitory concentration (FIC) testing of analogs with mupirocin against *S. aureus* UAMS-1.

	Dosed-Individually		Dosed-Combination		FIC Index ^b^
	Cpd MIC ^a^	Mup MIC ^a^	Cpd MIC ^a^	Mup MIC ^a^	
**RNP0007**	0.391	0.5	0.098	0.25	0.75
**RNP0008**	0.391	0.5	0.196	0.25	1
**RNP0009**	0.78	0.5	0.196	0.25	0.75
**RNP0010**	3.125	0.5	0.78	0.25	0.75
**RNP0011**	0.391	0.5	0.098	0.25	0.75
**RNP0012**	0.19	0.5	0.048	0.25	0.75
**RNP0013**	6.25	0.5	1.56	0.25	0.75
**RNP0014**	6.25	0.5	1.56	0.125	0.5
**RNP0015**	0.78	0.5	0.78	0.25	1.5
**RNP0016**	3.125	0.5	1.56	0.25	1
**RNP0017**	>500	0.5	-	-	-
**RNP0018**	>500	0.5	-	-	-

^a^ MIC values shown in µM. ^b^ FIC was calculated based on the formula summarized in the materials and methods. FIC ≤ 0.5 indicates synergistic activity, a value of 0.5 < FIC ≤ 1 indicates additive effect, a value between 1 and 4 indicated neutral effect and FIC greater than 4 indicated antagonism.

## Data Availability

The data presented in this study are available in the published article and supplementary information.

## Supplementary Materials

The following are available online at https://www.mdpi.com/article/10.3390/antibiotics10040369/s1, Table S1: Cell viability measurements of analogs against human HEP G2 cells, all analog synthesis and characterization data is provided in supplementary material.
