# Supplementary material for: Optimization of 2-Acylaminocycloalkylthiophene Derivatives for Activity against Staphylococcus aureus RnpA
Source: Antibiotics (Basel). 2021 Mar 31;10(4):369. doi: 10.3390/antibiotics10040369 (PMC8066339; doi:10.3390/antibiotics10040369)
Supplement: Supplementary file 1 [file antibiotics-10-00369-s001.pdf]

# Supplementary Material

**Table S1.** Cell viability measurements of analogs against human HEP G2 cells.

| Compound | 0 µM | 12.5 µM | 25 µM | 50 µM | 100 µM | 200 µM | 400 µM |
|---|---|---|---|---|---|---|---|
| Mitomycin C | 100±14 | | | | | | 54.8±1.8 |
| RNP0007 | 100±14 | 91.9±12.6 | 101.01±9.9 | 82.6±12.7 | 84.9±12.8 | 78.4±20.3 | 91.2±19.9 |
| RNP0008 | 100±11.8 | 85.9±19.4 | 100.6±13.6 | 83.9±13.6 | 75.7±6.8 | 70.1±12.01 | 60.1±8.3 |
| RNP0009 | 100±12.5 | 88.2±7.5 | 81.02±22.6 | 82.1±20.8 | 71.9±13.8 | 74.4±12.7 | 73.3±16.1 |
| RNP0010 | 100±12.5 | 92.2±25.8 | 95.8±3 | 93.5±11.4 | 74.4±12.8 | 63.5±9.2 | 61.9±13.6 |
| RNP0011 | 100±14 | 89.5±19.4 | 89.2±6.8 | 67.7±11.6 | 69.7±10.5 | 74.9±12.5 | 67.2±10.8 |
| RNP0012 | 100±14 | 98.6±12.4 | 95.5±14.4 | 68.4±10.9 | 66.7±5.5 | 62.8±7.2 | 62.6±3.7 |
| RNP0013 | 100±14 | 105.6±18 | 98.8±6.8 | 76.9±0.08 | 64.6±12.9 | 49.6±5.8 | 52.9±9.7 |
| RNP0014 | 100±14 | 75.9±16.4 | 84.4±14.6 | 79.1±11.2 | 76.7±13.02 | 58.1±13.4 | 59.6±17.01 |
| RNP0015 | 100±14 | 76.9±3.9 | 81.5±10.6 | 61.1±11.1 | 66.9±5.7 | 65.1±9.3 | 65.1±6.2 |
| RNP0016 | 100±14 | 86.2±11.1 | 89.1±15.2 | 69.2±7.5 | 64.1±5.2 | 61.1±11.9 | 57.9±7.1 |
| RNP0017 | 100±14 | 77.3±5.3 | 88.9±11.4 | 69.8±3.2 | 73.7±19.6 | 65.3±2.8 | 59.5±5.4 |
| RNP0018 | 100±14 | 69.3±9.8 | 76.9±12.3 | 68.4±9.8 | 61.6±17.5 | 64.8±8.1 | 69.8±24.3 |

**Analog Synthesis and characterization**

***N*-(3-chlorophenyl)-2-cyanoacetamide (1a):** Under Argon atmosphere, 3-chloroaniline (1.05 mL, 10.00 mmol) was dissolved in anhydrous DCM (20 mL), followed by addition of 2-cyanoacetic acid (0.85 g, 10.00 mmol) and EDC-HCl (2.30 g, 12.00 mmol), then stirred at room temperature overnight. The next day the mixture was concentrated *in vacuo*. The crude product was treated with 0.5 N HCl solution 200 mL, transferred to a separatory funnel and extracted with ethyl acetate. The organic phase was then washed with additional HCl (0.5 N solution), water, brine, and was then dried over $Na_2SO_4$ and concentrated under vacuum and the crude product was purified by manual silica gel column chromatography (Solvent A: hexanes, Solvent B: ethyl acetate; gradient 0 – 50% solvent B over 10 minutes) to afford **1a** (1.51 g, yield 77.6%) as white solid. $^1$H NMR (500 MHz, DMSO-$d_6$) δ 10.47 (s, 1H), 7.71 (d, *J* = 1.8 Hz, 1H), 7.38-7.29 (m, 2H), 7.12 (d, *J* = 7.8 Hz, 1H), 3.90 (s, 2H). MS (APCI) *m/z*: 195.0 (M+H)$^+$.

***N*-(3-fluorophenyl)-2-cyanoacetamide (1b):** Synthesized according to the same procedure as **1a** using 3-fluoroaniline (0.96 mL, 10.00 mmol), 2-cyanoacetic acid (0.85 g, 10.00 mmol) and EDC-HCl (2.30 g, 12.00 mmol) to yield **1b** (1.61 g, yield 90.4%) as white solid. $^1$H NMR (500 MHz, DMSO-$d_6$) δ 10.49 (s, 1H), 7.49 (dt, *J* = 11.5, 2.1 Hz, 1H), 7.33 (dd, *J* = 15.1, 8.1 Hz, 1H), 7.22 (dd, *J* = 8.2, 0.9 Hz, 1H), 6.92-6.86 (m, 1H), 3.89 (s, 2H). MS (APCI) m/z: 179.0 (M+H)$^+$.

***N*-(4-fluorophenyl)-2-cyanoacetamide (1c):** Synthesized according to the same procedure as **1a** using 4-fluoroaniline (0.96 mL, 10.00 mmol), 2-cyanoacetic acid (0.85 g, 10.00 mmol) and EDC-HCl (2.30 g, 12.00 mmol) to yield **1c** (1.28 g, yield 71.8%) as white solid. $^1$H NMR (500 MHz, DMSO-$d_6$) δ 10.33 (s, 1H), 7.52 (dt, *J* = 10.4, 4.2 Hz, 2H), 7.17-7.09 (m, 2H), 3.86 (s, 2H). MS (APCI) m/z: 179.0 (M+H)$^+$.

***N*-(3-methylphenyl)-2-cyanoacetamide (1d):** Synthesized according to the same procedure as **1a** using 3-methylaniline (1.05 mL, 10.00 mmol), 2-cyanoacetic acid (0.85 g, 10.00 mmol) and EDC-HCl (2.30 g, 12.00 mmol) to yield **1d** (1.33 g, yield 76.3%) as white solid. $^1H$ NMR (500 MHz, DMSO-$d_6$) δ 10.19 (s, 1H), 7.34 (s, 1H), 7.29 (d, *J* = 8.2 Hz, 1H), 7.17 (t, *J* = 7.8 Hz, 1H), 6.87 (d, *J* = 7.5 Hz, 1H), 3.85 (s, 2H), 2.24 (s, 3H). MS (APCI) m/z: 175.0 $(M+H)^+$.

***N*-(3-trifluoromethylphenyl)-2-cyanoacetamide (1e):** Synthesized according to the same procedure as **1a** using 3-trifluoromethylaniline (1.24 mL, 10.00 mmol), 2-cyanoacetic acid (0.85 g, 10.00 mmol) and EDC-HCl (2.30 g, 12.00 mmol) to yield **1e** (1.75 g, yield 76.8%) as white solid. $^1H$ NMR (500 MHz, DMSO-$d_6$) δ 10.63 (s, 1H), 7.99 (s, 1H), 7.69 (d, *J* = 8.2 Hz, 1H), 7.54 (t, *J* = 8.0 Hz, 1H), 7.41 (d, *J* = 7.7 Hz, 1H), 3.92 (s, 2H). MS (APCI,) m/z: 229.3 $(M+H)^+$.

***N*-(4-trifluoromethylphenyl)-2-cyanoacetamide (1f):** Synthesized according to the same procedure as **1a** using 4-trifluoromethylaniline (1.24 mL, 10.00 mmol), 2-cyanoacetic acid (0.85 g, 10.00 mmol) and EDC-HCl (2.30 g, 12.00 mmol) to yield **1f** (1.75 g, yield 76.8%) as white solid. (1.64 g, yield 71.8%) as white solid. $^1H$ NMR (500 MHz, DMSO-$d_6$) δ 10.64 (s, 1H), 7.72 (d, *J* = 8.6 Hz, 2H), 7.66 (d, *J* = 8.7 Hz, 2H), 3.94 (s, 2H). MS (APCI,) m/z: 229.3 $(M+H)^+$.

**2-amino-*N*-(3-chlorophenyl)-5,6,7,8-tetrahydro-4*H*-cyclohepta[b]thiophene-3-carboxamide (4a):** Under Argon atmosphere, **1a** (0.58 g, 3.00 mmol) were dissolved in toluene (30 mL), followed by the addition of the cycloheptanone (2.48 mL, 21.00 mmol), ammonium acetate (1.16 g, 15.00 mmol), glacial acetic acid (1.20 mL, 21.00 mmol), and $Na_2SO_4$ (2.13 g, 15.00 mmol). This reaction mixture was refluxed at 100 °C for 2 h. TLC indicated consumption of starting material and the formation of the desired Knoevenagel product **3a**. The mixture was then cooled to rt and treated with $NaHCO_3$ (5% aq) and transferred into a separatory funnel for extraction of product with ethyl acetate. The organic mixture was then washed with additional $NaHCO_3$ (5% aq), water, and saturated NaCl, and then then dried over $Na_2SO_4$ and concentrated in vacuo to afford **3a** as a brown oil, which was used for next step without any characterization or purification. The crude product **3a** (0.29 g, 1.00 mmol) was added to a vial under argon atmosphere and dissolved in ethanol (10 mL) then treated with molecular octasulfur (0.51 g, 2.00 mmol) and morpholine (0.28 mL, 3.00 mmol). The mixture was refluxed at 90 °C for 4 h. The mixture was then left to cool to room temperature. Upon reaching rt, the reaction mixture was filtered to remove excess precipitated inorganic sulfur materials. The filtrate was then concentrated in vacuo to afford crude product, then the crude product was purified by manual silica gel column chromatography (10% ethyl acetate/hexanes ~20 % ethyl acetate/hexane) to afford **4a** (0.15 g, 46.8%) as orange solid and interMS (ESI, LC-MS) m/z: 321.1 $(M+H)^+$.

**2-amino-*N*-(3-fluorophenyl)-5,6,7,8-tetrahydro-4*H*-cyclohepta[b]thiophene-3-carboxamide (4b):** Prepared according to the same procedure as **3a** using **1b** (0.54 g, 3.00 mmol) were dissolved in toluene (30 mL), followed by the addition of the cycloheptanone (2.48 mL, 21.00 mmol), ammonium acetate (1.16 g, 15.00 mmol), glacial acetic acid (1.20 mL, 21.00 mmol), and $Na_2SO_4$ (2.13 g, 15.00 mmol) to afford **3b** as a brown oil. The second step was performed as according to **4a** using **3b** (0.27 g, 1.0 mmol), octasulfur (0.51 g, 2.0 mmol), and morpholine (0.28 mL, 3.0 mmol) to produce **4b** (0.16 g, 52.6%) as orange solid. MS (ESI, LC-MS) m/z: 305.1 $(M+H)^+$.

**2-amino-*N*-(4-fluorophenyl)-5,6,7,8-tetrahydro-4*H*-cyclohepta[b]thiophene-3-carboxamide (4c):** Prepared according to the same procedure as **3a** using **1c** (0.54 g, 3.00 mmol) were dissolved in toluene (30 mL), followed by the addition of

the cycloheptanone (2.48 mL, 21.00 mmol), ammonium acetate (1.16 g, 15.00 mmol), glacial acetic acid (1.20 mL, 21.00 mmol), and $Na_2SO_4$ (2.13 g, 15.00 mmol) to afford **3c** as a brown oil. The second step was performed as according to **4a** using **3c** (0.27 g, 1.0 mmol), octasulfur (0.51 g, 2.0 mmol), and morpholine (0.28 mL, 3.0 mmol) to produce **4c** (0.18 g, 59.1%) as orange solid. MS (ESI, LC-MS) m/z: 305.1 $(M+H)^+$.

**2-amino-*N*-(3-methylphenyl)-5,6,7,8-tetrahydro-4*H*-cyclohepta[b]thiophene-3-carboxamide (4d):** Prepared according to the same procedure as **3a** using **1d** (0.52 g, 3.00 mmol) were dissolved in toluene (30 mL), followed by the addition of the cycloheptanone (2.48 mL, 21.00 mmol), ammonium acetate (1.16 g, 15.00 mmol), glacial acetic acid (1.20 mL, 21.00 mmol), and $Na_2SO_4$ (2.13 g, 15.00 mmol) to afford **3d** as a brown oil. The second step was performed as according to **4a** using **3d** (0.26 g, 1.0 mmol), octasulfur (0.51 g, 2.0 mmol), and morpholine (0.28 mL, 3.0 mmol) to produce **4d** (0.12 g, 39.9%) as orange solid. MS (ESI, LC-MS) m/z: 301.1 $(M+H)^+$.

**2-amino-*N*-(3-trifluoromethylphenyl)-5,6,7,8-tetrahydro-4*H*-cyclohepta[b]thiophene-3-carboxamide (4e):** Prepared according to the same procedure as **3a** using **1e** (0.69 g, 3.00 mmol) were dissolved in toluene (30 mL), followed by the addition of the cycloheptanone (2.48 mL, 21.00 mmol), ammonium acetate (1.16 g, 15.00 mmol), glacial acetic acid (1.20 mL, 21.00 mmol), and $Na_2SO_4$ (2.13 g, 15.00 mmol) to afford **3e** as a brown oil. The second step was performed as according to **4a** using **3e** (0.32 g, 1.0 mmol), octasulfur (0.51 g, 2.0 mmol), and morpholine (0.28 mL, 3.0 mmol) to produce **4e** (0.12 g, 34.0%) as orange solid. MS (ESI, LC-MS) m/z: 355.1 $(M+H)^+$.

**2-amino-*N*-(4-trifluoromethylphenyl)-5,6,7,8-tetrahydro-4*H*-cyclohepta[b]thiophene-3-carboxamide (4f):** Prepared according to the same procedure as **3a** using **1f** (0.69 g, 3.00 mmol) were dissolved in toluene (30 mL), followed by the addition of the cycloheptanone (2.48 mL, 21.00 mmol), ammonium acetate (1.16 g, 15.00 mmol), glacial acetic acid (1.20 mL, 21.00 mmol), and $Na_2SO_4$ (2.13 g, 15.00 mmol) to afford **3f** as a brown oil. The second step was performed as according to **4a** using **3f** (0.32 g, 1.0 mmol), octasulfur (0.51 g, 2.0 mmol), and morpholine (0.28 mL, 3.0 mmol) to produce **4f** (0.15 g, 42.0%) as orange solid. MS (ESI, LC-MS) m/z: 355.1 $(M+H)^+$.

**2-amino-*N*-(3-fluorophenyl)-4,5,6,7-tetrahydrobenzo[b]thiophene-3-carboxamide (4g):** Prepared according to the same procedure as **3a** using **1b** (0.54 g, 3.00 mmol) were dissolved in toluene (30 mL), followed by the addition of the cyclohexanone (2.18 mL, 21.00 mmol), ammonium acetate (1.16 g, 15.00 mmol), glacial acetic acid (1.20 mL, 21.00 mmol), and $Na_2SO_4$ (2.13 g, 15.00 mmol) to afford **3b** as a brown oil. The second step was performed as according to **4a** using **3g** (0.26 g, 1.0 mmol), octasulfur (0.51 g, 2.0 mmol), and morpholine (0.28 mL, 3.0 mmol) to produce **4g** (0.064 g, 22.0%) as orange solid. MS (ESI, LC-MS) m/z: 291.1 $(M+H)^+$.

**2-amino-*N*-(4-fluorophenyl)-4,5,6,7-tetrahydrobenzo[b]thiophene-3-carboxamide (4h):** Prepared according to the same procedure as **3a** using **1c** (0.54 g, 3.00 mmol) were dissolved in toluene (30 mL), followed by the addition of the cyclohexanone (2.18 mL, 21.00 mmol), ammonium acetate (1.16 g, 15.00 mmol), glacial acetic acid (1.20 mL, 21.00 mmol), and $Na_2SO_4$ (2.13 g, 15.00 mmol) to afford **3h** as a brown oil. The second step was performed as according to **4a** using **3h** (0.26 g, 1.0 mmol), octasulfur (0.51 g, 2.0 mmol), and morpholine (0.28 mL, 3.0 mmol) to produce **4h** (0.080 g, 27.6%) as orange solid. MS (ESI, LC-MS) m/z: 291.1 $(M+H)^+$

**2-amino-*N*-(4-trifluoromethylphenyl)-4,5,6,7-tetrahydrobenzo[b]thiophene-3-carboxamide (4i):** Prepared according

to the same procedure as **3a** using **1f** (0.69 g, 3.00 mmol) were dissolved in toluene (30 mL), followed by the addition of the cyclohexanone (2.18 mL, 21.00 mmol), ammonium acetate (1.16 g, 15.00 mmol), glacial acetic acid (1.20 mL, 21.00 mmol), and $Na_2SO_4$ (2.13 g, 15.00 mmol) to afford **3i** as a brown oil. The second step was performed as according to **4a** using **3i** (0.31 g, 1.0 mmol), octasulfur (0.51 g, 2.0 mmol), and morpholine (0.28 mL, 3.0 mmol) to produce **4i** (0.060 g, 17.7%) as orange solid. MS (ESI, LC-MS) m/z: 341.1 $(M+H)^+$.

**2-amino-*N*-(3-trifluoromethylphenyl)-4,5,6,7-tetrahydrobenzo[b]thiophene-3-carboxamide (4j):** Prepared according to the same procedure as **3a** using **1e** (0.69 g, 3.00 mmol) were dissolved in toluene (30 mL), followed by the addition of the cyclohexanone (2.18 mL, 21.00 mmol), ammonium acetate (1.16 g, 15.00 mmol), glacial acetic acid (1.20 mL, 21.00 mmol), and $Na_2SO_4$ (2.13 g, 15.00 mmol) to afford **3j** as a brown oil. The second step was performed as according to **4a** using **3j** (0.31 g, 1.0 mmol), octasulfur (0.51 g, 2.0 mmol), and morpholine (0.28 mL, 3.0 mmol) to produce **4j** (0.068 g, 20.1%) as orange solid. MS (ESI, LC-MS) m/z: 341.3 $(M+H)^+$.

***N*-(3-chlorophenyl)-2-(2,2,2-trifluoroacetamido)-5,6,7,8-tetrahydro-4*H*-cyclohepta[b]thiophene-3-carbox-amide (RNP0007).** Under argon atmosphere, **4a** (0.064 g, 0.20 mmol) was dissolved in anhydrous DCM (2 mL), treated with triethylamine (0.036 mL, 0.26 mmol) in DCM (0.5 mL) and trifluoroacetic anhydride (0.033 mL, 0.26 mmol) in anhydrous DCM (0.5 mL). The reaction was stirred at rt, until LC-MS confirmed consumption of starting material and formation of product. The mixture was then transferred into a separatory funnel with ethyl acetate. The organic layer was then washed with water, NaCl (satd aq), dried over $Na_2SO_4$, and then concentrated in vacuo to yield yellow solid, then the crude product was purified by manual Silica gel column Chromatography (0% Ethyl acetate/Hexane ~5 % Ethyl acetate/Hexane) to afford **RNP0007** (0.039 g, 47.0% yield) as pale yellow solid. $^1H$ NMR (500 MHz, $CDCl_3$) $\delta$ 11.92 (s, 1H), 7.66 (d, $J$ = 1.7 Hz, 1H), 7.40 (dd, $J$ = 8.1, 0.9 Hz, 1H), 7.31 (t, $J$ = 8.0 Hz, 2H), 7.19-7.16 (m, 1H), 2.97-2.93 (m, 2H), 2.84-2.80 (m, 2H), 1.92 (dt, $J$ = 11.7, 6.0 Hz, 2H), 1.76 (qd, $J$ = 11.1, 7.9 Hz, 4H). $^{13}C$ NMR (126 MHz, $CDCl_3$) $\delta$ 164.1, 153.3, 138.8, 137.8, 135.0, 134.9, 133.0, 130.2, 125.2, 120.4, 120.1, 118.3, 31.5, 28.9, 28.7, 27.3, 27.0. MS (APCI) m/z: 833.0 $(2M+H)^+$. HPLC, retention time = 13.318 min, purity = 95.9%.

***N*-(3-fluorophenyl)-2-(2,2,2-trifluoroacetamido)-5,6,7,8-tetrahydro-4*H*-cyclohepta[b]thiophene-3-carbox-amide (RNP0008).** This molecule was prepared similar to RNP0007 using **4b** (0.061 g, 0.20 mmol), triethylamine (0.036 mL, 0.26 mmol), and trifluoroacetic anhydride (0.033 mL, 0.26 mmol) to produce **RNP0008** (0.013 g, 15.8% yield) as pale yellow solid. $^1H$ NMR (500 MHz, $CDCl_3$) $\delta$ 11.93 (s, 1H), 7.53 (d, $J$ = 10.5 Hz, 1H), 7.34 (q, $J$ = 8.2 Hz, 2H), 7.18 (d, $J$ = 8.0 Hz, 1H), 6.89 (tt, $J$ = 9.3, 4.7 Hz, 1H), 2.98-2.93 (m, 2H), 2.85-2.80 (m, 2H), 1.93 (dt, $J$ = 11.6, 6.0 Hz, 2H), 1.76 (ddd, $J$ = 14.3, 8.4, 5.7 Hz, 4H). $^{13}C$ NMR (126 MHz, $CDCl_3$) $\delta$ 164.0, 153.6, 138.8, 138.2, 135.0, 133.0, 130.3, 130.3, 120.1, 115.5, 112.0, 111.8, 108.0, 107.8, 31.5, 28.9, 28.7, 27.3, 27.0. MS (APCI) m/z: 801.2 $(2M+H)^+$. HPLC, retention time = 12.983 min, purity = 95.1%.

***N*-(4-fluorophenyl)-2-(2,2,2-trifluoroacetamido)-5,6,7,8-tetrahydro-4*H*-cyclohepta[b]thiophene-3-carbox-amide (RNP0009).** This molecule was prepared similar to RNP0007 using **4c** (0.061 g, 0.20 mmol), triethylamine (0.036 mL, 0.26 mmol), and trifluoroacetic anhydride (0.033 mL, 0.26 mmol) to produce **RNP0009** (0.012 g, 12.0% yield) as pale yellow solid. $^1H$ NMR (500 MHz, $CDCl_3$) $\delta$ 11.99 (s, 1H), 7.54-7.48 (m, 2H), 7.28 (d, $J$ = 8.3 Hz, 1H), 7.10 (t, $J$ = 8.6 Hz, 2H), 2.95 (s, 2H), 2.82 (dd, $J$ = 6.5, 4.2 Hz, 2H), 1.97-1.87 (m, 2H), 1.76 (d, $J$ = 5.0 Hz, 4H). $^{13}C$ NMR (126 MHz, $CDCl_3$) $\delta$ 164.1, 158.9,

153.5, 153.2, 138.6, 134.9, 133.0, 132.5, 122.5, 122.4, 120.1, 116.0, 115.9, 109.2, 31.5, 28.9, 28.7, 27.3, 27.0. MS (APCI) m/z: 801.2 $(2M+H)^+$. HPLC, retention time = 12.860 min, purity = 95.1%.

***N*-(3-methylphenyl)-2-(2,2,2-trifluoroacetamido)-5,6,7,8-tetrahydro-4*H*-cyclohepta[b]thiophene-3-carbox-amide (RNP0010).** This molecule was prepared similar to RNP0007 using **4d** (0.060 g, 0.20 mmol), triethylamine (0.036 mL, 0.26 mmol), and trifluoroacetic anhydride (0.033 mL, 0.26 mmol) to produce **RNP0010** (0.029 g, 36.0 % yield) as pale yellow solid. $^1$H NMR (500 MHz, $CDCl_3$) $\delta$ 12.01 (s, 1H), 7.36 (s, 1H), 7.34 (d, $J$ = 8.2 Hz, 1H), 7.29 (d, $J$ = 7.6 Hz, 1H), 7.02 (d, $J$ = 7.4 Hz, 1H), 2.98-2.93 (m, 2H), 2.81 (dd, $J$ = 6.7, 4.3 Hz, 2H), 2.39 (s, 3H), 1.92 (dt, $J$ = 11.6, 6.0 Hz, 2H), 1.75 (dd, $J$ = 13.7, 8.4 Hz, 4H). $^{13}$C NMR (126 MHz, $CDCl_3$) $\delta$ 164.0, 153.5, 153.2, 139.2, 138.4, 136.5, 134.8, 133.2, 129.0, 126.1, 121.1, 120.5, 117.6, 31.5, 28.9, 28.7, 27.4, 27.0, 21.4. MS (APCI,) m/z: 793.3 $(2M+H)^+$. HPLC, retention time = 13.164 min, purity = 98.3%.

***N*-(3-trifluoromethylphenyl)-2-(2,2,2-trifluoroacetamido)-5,6,7,8-tetrahydro-4*H*-cyclohepta[b]thiophene-3-carbox-amide (RNP0011).** This molecule was prepared similar to RNP0007 using **4e** (0.071 g, 0.20 mmol), triethylamine (0.036 mL, 0.26 mmol), and trifluoroacetic anhydride (0.033 mL, 0.26 mmol) to produce **RNP0011** (0.011 g, 12.0 % yield) as pale yellow solid. $^1$H NMR (500 MHz, $CDCl_3$) $\delta$ 11.90 (s, 1H), 7.81 (s, 1H), 7.78 (d, $J$ = 8.1 Hz, 1H), 7.52 (t, $J$ = 7.9 Hz, 1H), 7.46 (d, $J$ = 7.8 Hz, 1H), 7.39 (s, 1H), 3.00-2.94 (m, 2H), 2.83 (dd, $J$ = 6.7, 4.2 Hz, 2H), 1.93 (dt, $J$ = 11.7, 6.0 Hz, 2H), 1.80-1.72 (m, 4H). $^{13}$C NMR (126 MHz, $CDCl_3$) $\delta$ 164.2, 156.2, 145.8, 139.0, 137.2, 135.1, 132.9, 129.8, 123.5, 122.5, 121.7, 119.9, 118.9, 117.1, 34.4, 29.0, 28.7, 27.3, 27.0. MS (APCI,) m/z: 451.1 $(M+H)^+$. HPLC, retention time = 13.327 min, purity = 97.1%.

***N*-(4-trifluoromethylphenyl)-2-(2,2,2-trifluoroacetamido)-5,6,7,8-tetrahydro-4*H*-cyclohepta[b]thiophene-3-carbox-amide (RNP0012).** This molecule was prepared similar to RNP0007 using **4f** (0.071 g, 0.20 mmol), triethylamine (0.036 mL, 0.26 mmol), and trifluoroacetic anhydride (0.033 mL, 0.26 mmol) to produce **RNP0012** (0.019 g, 20.7 % yield) as pale yellow solid. $^1$H NMR (500 MHz, $CDCl_3$) $\delta$ 11.88 (s, 1H), 7.69 (d, $J$ = 8.6 Hz, 2H), 7.65 (d, $J$ = 8.7 Hz, 2H), 7.44 (s, 1H), 2.99-2.94 (m, 2H), 2.82 (dd, $J$ = 6.7, 4.3 Hz, 2H), 1.97-1.90 (m, 2H), 1.81-1.72 (m, 4H). $^{13}$C NMR (126 MHz, $CDCl_3$) $\delta$ 164.2, 153.6, 153.3, 153.2, 139.8, 139.0, 135.1, 133.0, 126.4, 126.4, 120.0, 31.3, 28.9, 28.7, 27.3, 27.0. MS (APCI) m/z: 451.2 $(M+H)^+$. HPLC, retention time = 13.342 min, purity = 95.9%.

***N*-(3-fluorophenyl)-2-(2,2,2-trifluoroacetamido)-4,5,6,7-tetrahydrobenzo[b]thiophene-3-carboxamide (RNP0013).** This molecule was prepared similar to RNP0007 using **4g** (0.058 g, 0.20 mmol), triethylamine (0.036 mL, 0.26 mmol), and trifluoroacetic anhydride (0.033 mL, 0.26 mmol) to produce **RNP0013** (0.022 g, 28.3 % yield) as pale yellow solid. $^1$H NMR (500 MHz, $CDCl_3$) $\delta$ 13.09 (s, 1H), 7.74 (s, 1H), 7.54 (dt, $J$ = 10.6, 2.2 Hz, 1H), 7.33 (td, $J$ = 8.2, 6.5 Hz, 1H), 7.19 (dd, $J$ = 8.1, 1.3 Hz, 1H), 6.89 (td, $J$ = 8.3, 2.0 Hz, 1H), 2.89 (t, $J$ = 5.7 Hz, 2H), 2.77 (t, $J$ = 5.9 Hz, 2H), 1.99-1.88 (m, 4H). $^{13}$C NMR (126 MHz, $CDCl_3$) $\delta$ 164.0, 163.9, 161.9, 153.4, 143.3, 138.2, 138.1, 130.8, 130.3, 130.2, 126.9, 116.6, 115.8, 112.0, 111.8, 108.4, 108.1, 26.6, 24.4, 22.7, 22.3. MS (APCI) m/z: 387.0 $(M+H)^+$. HPLC, retention time = 12.987 min, purity = 97.9%.

***N*-(4-fluorophenyl)-2-(2,2,2-trifluoroacetamido)-4,5,6,7-tetrahydrobenzo[b]thiophene-3-carboxamide (RNP0014).** This molecule was prepared similar to RNP0007 using **4h** (0.058 g, 0.20 mmol), triethylamine (0.036 mL, 0.26 mmol), and trifluoroacetic anhydride (0.033 mL, 0.26 mmol) to produce **RNP0014** (0.018 g, 24.8 % yield) as pale yellow solid. $^1$H NMR (500 MHz, $CDCl_3$) $\delta$ 13.16 (s, 1H), 7.67 (s, 1H), 7.55-7.48 (m, 2H), 7.11-7.05 (m, 2H), 2.89 (t, $J$ = 5.6 Hz, 2H), 2.77 (t, $J$

= 5.8 Hz, 2H), 2.00-1.87 (m, 4H). $^{13}C$ NMR (126 MHz, $CDCl_3$) $\delta$ 164.1, 160.9, 159.0, 153.7, 153.4, 143.0, 132.5, 130.7, 127.0, 123.0, 122.9, 116.6, 116.0, 115.8, 26.6, 24.4, 22.7, 22.3. MS (APCI) m/z: 387.1 $(M+H)^+$. HPLC, retention time = 13.131 min, purity = 95.2%.

***N*-(4-trifluoromethylphenyl)-2-(2,2,2-trifluoroacetamido)-4,5,6,7-tetrahydrobenzo[b]thiophene-3-carboxamide (RNP0015).** This molecule was prepared similar to RNP0007 using **4i** (0.068 g, 0.20 mmol), triethylamine (0.036 mL, 0.26 mmol), and trifluoroacetic anhydride (0.033 mL, 0.26 mmol) to produce **RNP0015** (0.021 g, 23.6 % yield) as pale yellow solid. $^1H$ NMR (500 MHz, $CDCl_3$) $\delta$ 13.03 (s, 1H), 7.83 (s, 1H), 7.71 (d, *J* = 8.5 Hz, 2H), 7.64 (d, *J* = 8.6 Hz, 2H), 2.91 (t, *J* = 5.6 Hz, 2H), 2.77 (t, *J* = 5.8 Hz, 2H), 1.99-1.88 (m, 4H).$^{13}C$ NMR (126 MHz, $CDCl_3$) $\delta$ 164.2, 153.8, 153.5, 143.6, 139.8, 130.9, 127.0, 126.9, 126.4, 126.4, 120.3, 116., 114.4, 26.6, 24.4, 22.6, 22.3. MS (APCI) m/z: 437.0 $(M+H)^+$. HPLC, retention time = 13.426 min, purity = 95.7%.

***N*-(3-trifluoromethylphenyl)-2-(2,2,2-trifluoroacetamido)-4,5,6,7-tetrahydrobenzo[b]thiophene-3-carboxamide (RNP0016).** This molecule was prepared similar to RNP0007 using **4j** (0.068 g, 0.20 mmol), triethylamine (0.036 mL, 0.26 mmol), and trifluoroacetic anhydride (0.033 mL, 0.26 mmol) to produce **RNP0016** (0.022 g, 24.3 % yield) as pale yellow solid. $^1H$ NMR (500 MHz, $CDCl_3$) $\delta$ 13.05 (s, 1H), 7.81 (d, *J* = 6.7 Hz, 3H), 7.52 (t, *J* = 7.9 Hz, 1H), 7.45 (d, *J* = 7.7 Hz, 1H), 2.92 (t, *J* = 5.6 Hz, 2H), 2.77 (t, *J* = 5.9 Hz, 2H), 2.01-1.88 (m, 4H). $^{13}C$ NMR (126 MHz, $CDCl_3$) $\delta$ 164.2, 153.8, 153.5, 143.5, 137.2, 131.7, 131.4, 130.8, 129.7, 126.9, 124.7, 123.9, 121.7, 117.4, 116.4, 26.6, 24.4, 22.6, 22.3. MS (APCI) m/z: 436.9 $(M+H)^+$. HPLC, retention time = 13.401 min, purity = 95.3%.

***N*-(3-chlorophenyl)-2-(2,2-difluoroacetamido)-5,6,7,8-tetrahydro-4*H*-cyclohepta[b]thiophene-3-carboxamide (RNP0017).** Under Argon atmosphere, a solution of the 2,2-difluoroacetic acid (0.015 g, 0.15 mmol), 4-DMAP (0.002 g, 0.012 mmol), and EDC-HCl (0.035 g, 0.19 mmol) in anhydrous DCM (2 mL) was stirred at rt for 30 min. A solution of **4a** (40.00 mg, 124.7 μmol) in Anhydrous DCM (2 mL) was added into the previous reaction mixture, then stirred at room temperature overnight; The reactions was stirred at rt, until LC-MS confirmed consumption of starting material and formation of product. The mixture was then transferred into a separatory funnel with ethyl acetate. The organic layer was then washed with water, NaCl (satd aq), dried over $Na_2SO_4$, and then concentrated in vacuo to yield yellow solid, then the crude product was purified by manual Silica gel column Chromatography (0% Ethyl acetate/Hexane ~5 % Ethyl acetate/Hexane) to afford **RNP0017** (0.016 g, 32.2% yield) as pale yellow solid. $^1H$ NMR (500 MHz, $CDCl_3$) $\delta$ 11.58 (s, 1H), 7.67 (d, *J* = 1.7 Hz, 1H), 7.43-7.39 (m, 1H), 7.33 -7.28 (m, 2H), 7.16 (dd, *J* = 8.0, 0.9 Hz, 1H), 6.07 (t, *J* = 54.1 Hz, 1H), 2.95-2.91 (m, 2H), 2.82-2.77 (m, 2H), 1.92 (dt, *J* = 11.6, 6.0 Hz, 2H), 1.80-1.70 (m, 4H). $^{13}C$ NMR (126 MHz, $CDCl_3$) $\delta$ 164.02, 159.06, 158.86, 139.09, 138.00, 134.81, 134.32, 132.84, 130.13, 125.00, 120.31, 119.86, 118.20, 109.86, 107.85, 105.83, 31.55, 28.90, 28.66, 27.36, 27.09. MS (APCI) m/z: 399.0 $(M+H)^+$. HPLC, retention time = 12.800 min, purity = 96.0%.

***N*-(3-chlorophenyl)-2-(2-fluoroacetamido)-5,6,7,8-tetrahydro-4*H*-cyclohepta[b]thiophene-3-carboxamide (RNP0018).** Under Argon atmosphere, a solution of the 2-fluoroacetic acid (0.012 g, 0.15 mmol), 4-DMAP (0.002 g, 0.012 mmol), and EDC-HCl (0.036 g, 0.187 mmol) in anhydrous DCM (2 mL) was stirred at rt for 30 min. A solution of **12** (40.00 mg, 124.7 μmol) in anhydrous DCM (2 mL) was added into the previous reaction mixture, then stirred at room temperature overnight; The reactions was stirred at rt, until LC-MS confirmed consumption of starting material and formation of product.

The mixture was then transferred into a separatory funnel with ethyl acetate. The organic layer was then washed with water, NaCl (satd aq), dried over $Na_2SO_4$, and then concentrated in vacuo to yield yellow solid, then the crude product was purified by manual Silica gel column Chromatography (0% Ethyl acetate/Hexane ~5 % Ethyl acetate/Hexane) to afford **RNP0018** (0.020 g, 42.1% yield) as pale yellow solid. $^1$H NMR (500 MHz, $CDCl_3$) $\delta$ 11.24 (s, 1H), 7.70 (dd, $J$ = 9.8, 7.8 Hz, 1H), 7.43-7.39 (m, 1H), 7.32-7.27 (m, 2H), 7.17-7.13 (m, 1H), 5.00 (d, $J$ = 23.2 Hz, 1H), 4.91 (d, $J$ = 23.5 Hz, 1H), 2.94-2.89 (m, 2H), 2.82- 2.77 (m, 2H), 1.91 (dt, $J$ = 11.6, 6.0 Hz, 2H), 1.74 (dt, $J$ = 11.2, 5.6 Hz, 4H). $^{13}$C NMR (126 MHz, $CDCl_3$) $\delta$ 164.0, 158.9, 139.5, 138.2, 134.8, 133.4, 132.6, 130.1, 125.1, 124.8, 120.1, 120.1, 118.0, 117.9, 80.4, 78.9, 31.7, 28.9, 28.7, 27.4, 27.2. MS (APCI) m/z: 381.0 $(M+1)^+$. HPLC, retention time = 12.640 min, purity = 96.2%.
